# Redefining Chronic Toxoplasmosis—A T Cell Exhaustion Perspective

**DOI:** 10.1371/journal.ppat.1002903

**Published:** 2012-10-11

**Authors:** Rajarshi Bhadra, Imtiaz A. Khan

**Affiliations:** Department of Microbiology, Immunology and Tropical Medicine, George Washington University, Washington, D.C., United States of America; University of Wisconsin Medical School, United States of America

## Chronic Toxoplasmosis and CD8 Exhaustion

**Figure 1:**
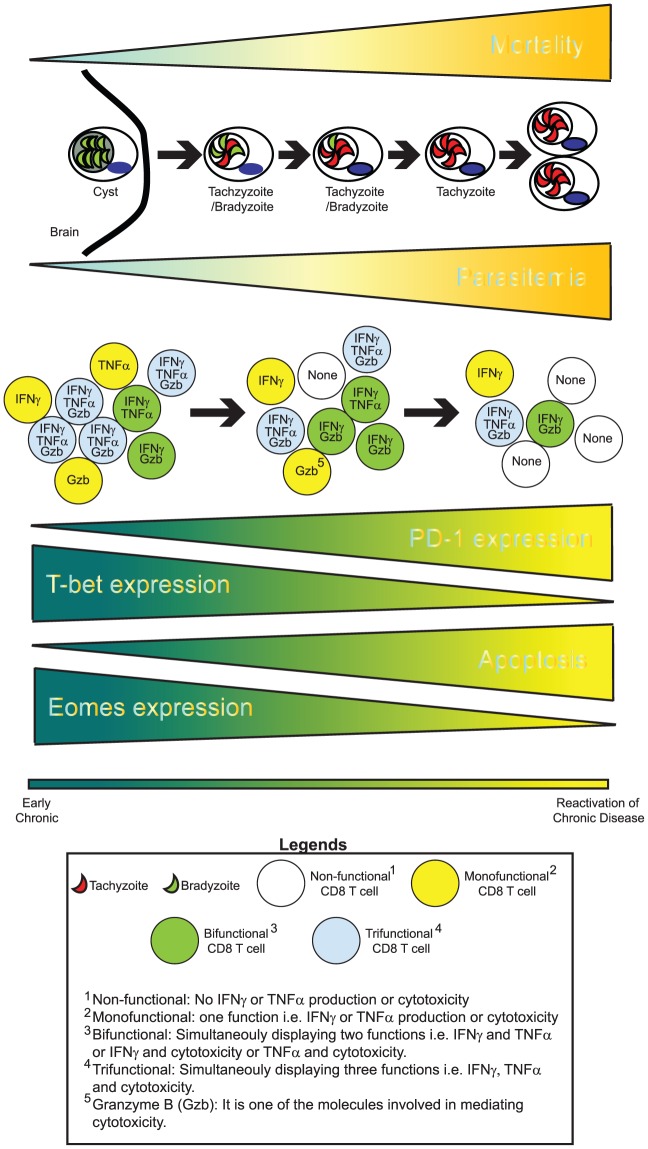
CD8 exhaustion during chronic toxoplasmosis. During the later phases of chronic toxoplasmosis, concomitant with elevated mortality, encysted bradyzoites (parasites in green) undergo reactivation. This process involves *T. gondii* parasites undergoing stage conversion from quiescent, slow-replicating bradyzoites to highly motile, fast-replicating tachyzoites (parasites shown in red). Parasite reactivation results in disease recrudescence and eventual host mortality from toxoplasmic encephalitis. Cytokines such as IFNγ and TNFα as well cytotoxicity play an important role in controlling *T. gondii* infection. During chronic toxoplasmosis, CD8 T cells progressively fail to produce optimal IFNγ or TNFα and mediate cytotoxicity. Additionally, their ability to simultaneously exhibit all these functions (polyfunctionality) is lost. This is significant considering that improved pathogen control in HIV^+^ nonprogressors has been shown to be strongly correlated with CD8 polyfunctionality. This loss of CD8 polyfunctionality is concomitant with elevated PD-1 expression on CD8 T cells, decreased expression of transcription factors T-bet and Eomes, and elevated CD8 apoptosis. It is likely that an altered transcriptional profile in combination with PD-1–mediated apoptosis results in loss of polyfunctional CD8 T cells over time. This phenomenon involving graded attrition of CD8 functionality is referred to as CD8 exhaustion.

Toxoplasmosis caused by *Toxoplasma gondii (T. gondii)*, an obligate intercellular protozoan, is considered to be a leading cause of foodborne mortality in the United States (www.cdc.gov). Even in the post-HAART era, fatal toxoplasmic encephalitis (TE) due to reactivation of chronic *Toxoplasma* infection remains a major problem in *Toxoplasma*-seropositive AIDS patients in developing countries [1]. In warm-blooded intermediate hosts (including humans), the parasite undergoes stage conversion between the rapidly proliferating tachyzoite, which is considered to be responsible for acute toxoplasmosis, and the relatively quiescent, slowly replicating, encysted bradyzoite that can persist for life. However, in the immunocompromised such as AIDS patients, the parasite converts from a bradyzoite to a tachyzoite stage, leading to TE [2]. Similarly, repeated reactivations can also occur in congenitally infected individuals [3]. Tachyzoite–bradyzoite interconversion is believed to play a central role not only in establishing the chronic infection but also in disease recrudescence [2]. However, factors responsible for the reactivation of chronic infection in vivo remain poorly understood [2], [4]. Studies in murine models of chronic toxoplasmosis have demonstrated that CD8 T cells are pivotal for long-term protection [3]. Paradoxically, despite a robust CD8 T cell response during the acute phase of infection, long-term immunity against this pathogen is compromised in susceptible mouse strains, leading to reactivation and host mortality. Differential susceptibility to *T. gondii* reactivation in AIDS patients was also noted in a study conducted during the pre-HAART era, which reported that only 30% of AIDS patients with low CD4 T cell count and *Toxoplasma* seropositivity, who were not on effective prophylaxis, developed reactivated toxoplasmosis [5]. Why does a modest subset of this high-risk group develop TE? Considering that memory CD8 T cells can persist for a lifetime and can mediate protective recall responses upon antigen reencounter in other infectious diseases [6], it remains to be addressed whether this differential outcome is a consequence of potential attrition of *T. gondii*–specific memory CD8 T cells due to genetic polymorphisms or other microenvironment-associated factors. Recent studies from our group, which utilized a susceptible mouse model (C57BL/6), have demonstrated that CD8 T cells during the later phase of chronic toxoplasmosis exhibit progressive attrition of functionality, increased apoptosis, and poor recall response along with elevated expression of PD-1, an inhibitory receptor-a phenomenon referred to as CD8 exhaustion [7]. Concomitant with graded CD8 exhaustion, parasites undergo reactivation resulting in the mortality of the infected host (Figure 1). While the paradigm of CD8 exhaustion has been extensively explored in chronic viral models, it is just beginning to unfold in parasitic infections. Unlike chronic viral models of CD8 exhaustion, which are characterized by persistent high viremia, the *T. gondii* model represents a unique situation where, despite initial control of parasitemia, CD8 T cells eventually become exhausted [7], [8]. Considering that current drugs against *T. gondii* are toxic and inefficacious against the encysted bradyzoite stage of the parasite [2], [3], a thorough understanding of T cell exhaustion during chronic toxoplasmosis is critical for the development of improved immunotherapeutics against this pathogen. Significantly, our laboratory has demonstrated that a blockade of PD-1 interaction with its receptor PD-L1, via anti-PD-L1 antibody treatment of chronically infected animals, not only reinvigorates CD8 response and controls parasite reactivation but also prevents host mortality [7].

## CD8 Polyfunctionality

Polyfunctionality, i.e. the capacity of a single T cell to display multiple functions, is one of the hallmarks of robust CD8 response. This is emphasized by the observation that virus-specific polyfunctional CD8 T cell response, rather than absolute CD8 number, correlates with superior viral control in HIV-infected nonprogressors [9]. Examination of the polyfunctional (IFNγ, Granzyme B, TNFα, and IL-2) CD8 subset in *T. gondii*–infected animals revealed that concomitant with *Toxoplasma* reactivation and elevated CD8-mediated PD-1 expression, the frequency of polyfunctional CD8 T cells declined sharply both in spleen and brain [7], [10]. Since T-box factors T-bet and Eomesodermin play an important role in mediating CD8 functionality [11], investigation of the transcriptional profile revealed a decrease in both T-bet and Eomes expression in CD8 T cells. Significantly, treatment of chronically infected mice with αPD-L1 not only rescued bi-functional and tri-functional CD8 response but also augmented T-bet and Eomes expression [7], [10]. Although αPD-L1 therapy revived CD8 polyfunctionality, IL-2 expression remained refractory to this treatment [10]. Apart from the per-cell decrease of functionality, preferential apoptosis of polyfunctional CD8 T cells could also account for the attrition of polyfunctional response during chronic toxoplasmosis. In agreement with this notion, polyfunctional memory CD8 T cells preferentially expressed high PD-1 levels which rendered them susceptible to apoptosis [12]. Combined, this suggests that an altered transcriptional profile and preferential apoptosis result in attrition of polyfunctional CD8 response during chronic toxoplasmosis. Considering that T cell fate imprinting can occur as early as the first cell division [13], that raises the following question: Are CD8 T cells programmed to become dysfunctional at the very beginning? Preliminary studies using drug treatment demonstrate that early rather the than late intervention is critical for maintaining optimal CD8 polyfunctionality and preventing high PD-1 expression [7]. This suggests that high antigen burden or inflammation (including anti-inflammatory factors) or both during the acute phase of infection may be responsible for development of CD8 exhaustion during the late chronic stage of infection.

## Skewed Memory CD8 Development

Memory CD8 T cells are critical for long-term protection against intracellular pathogens [14]. During chronic toxoplasmosis, the majority of *T. gondii*–specific polyfunctional CD8 T cells exhibit cardinal markers of memory phenotype CD44 and CD127 [12]. Yet in contrast to acute infection models, these memory CD8 T cells exhibit high PD-1 expression [12]. Robust recall response upon secondary challenge is one of the hallmarks of potent CD8 memory development [15]. However, CD8 T cells during the later phases of chronic response are deficient in this regard [7]. Further, subset-specific analysis demonstrated that PD-1–expressing memory phenotype CD8 T cells expressed high levels of CD43, a marker of effector CD8 lineage [12]. Incidentally, CD43^hi^ CD8 T cells have been shown to elicit a poor recall response in a Sendai virus model [16]. Despite the expression of effector lineage–associated marker CD43, PD-1^hi^ CD8 T cells expressed relatively high levels of anti-apoptotic molecule Bcl-2, a hallmark of conventional memory CD8 T cells [12], [17]. Taken together this suggests that while PD-1^hi^ CD8 T cells display both memory and effector lineage characteristics, they represent a phenotype distinct from conventional memory and effector CD8 T cells generated in nonchronic models of infection. Considering that IL-7 and IL-15 are critical for homeostasis of memory CD8 T cells, it will be important to determine if this skewed phenotype results in poor responsiveness to these cytokines and whether immunotherapy with these cytokines is able to rescue or prevent CD8 exhaustion [3], [18].

## Role of Positive Costimulatory Signals during the Rescue

A recent study from our laboratory demonstrated that a blockade of negative signals such as PD-1-PD-L1 alone is insufficient for the rescue of exhausted CD8 T cells, and positive costimulatory signals, namely the CD40-CD40L pathway, play a pivotal role during this process [10]. Using a mixed bone marrow chimera (Wild-type:CD40−/−) approach, our group demonstrated that CD40 expression directly on CD8 T cells has a minimal impact on their functionality or development or PD-1 expression [10]. However, CD40 sufficiency on CD8 T cells plays a critical role during αPD-L1–mediated rescue of CD8 response in terms of optimal expansion and polyfunctional CD8 response development. Significantly, CD8-extrinsic CD40 signaling is also a major contributor to the process. In absence of CD40-CD40L signaling, CD40-deficient CD4 T cells in αPD-L1–treated chimeras do not develop a robust T follicular helper (Tfh) cell response or express copious IL-21 [10]. Incidentally, the role of IL-21 in preventing CD8 exhaustion is well established in chronic viral models [8]. It is likely that both CD40L-expressing antigen presenting cells (APC) and CD4 T cells interact with CD40^+^ CD8 T cells to mediate CD8 rescue in αPD-L1–treated mice. In future studies, it will be important to decipher the differential contribution of Tfh and APCs to the rescue of exhausted CD8 T cells.

## Future Perspectives

Current studies on CD8 exhaustion provide a new insight into chronic toxoplasmosis and bear the promise for improved immunotherapeutics against this pathogen. However several issues remain unaddressed. Although αPD-L1 treatment prevented the mortality of chronically infected animals, this was only achieved with prolonged antibody therapy. Extended αPD-L1 treatment carries the risk of developing autoimmune reactions. Additionally, αPD-L1 treatment was only efficacious on PD-1^int^ CD8 T cells, and PD-1^hi^ CD8 T cells remained refractory to its anti-apoptotic effects [7]. This potentially raises the question of whether PD-1^hi^ cells coexpress other inhibitory receptors (Tim3, Lag3, 2B4, etc.), which renders them insensitive to αPD-L1 therapy [19]. Another issue that needs to be addressed: Are strains such as Balb/C resistant to *Toxoplasma* because they do not develop exhaustion? If so, a microarray analysis of *T. gondii*–specific CD8 T cells from resistant and susceptible mice stains will be important in unraveling novel pathways and molecules that may be involved in this divergent outcome to *Toxoplasma* challenge. Using such an approach will permit us to develop better immunotherapy not only against *Toxoplasma* but also other chronic infectious diseases and cancers.
